# Vitamin D supplement on prevention of fall and fracture: A Meta-analysis of Randomized Controlled Trials

**DOI:** 10.1097/MD.0000000000021506

**Published:** 2020-08-21

**Authors:** Saran Thanapluetiwong, Api Chewcharat, Kullaya Takkavatakarn, Kearkiat Praditpornsilpa, Somchai Eiam-Ong, Paweena Susantitaphong

**Affiliations:** a Division of Geriatric Medicine, Department of Medicine, Ramathibodi Hospital, Mahidol University; b Division of Nephrology, Department of Medicine, Faculty of Medicine, King Chulalongkorn Memorial Hospital; c Research Unit for Metabolic Bone Disease in CKD Patients, Faculty of Medicine, Chulalongkorn University, Bangkok, Thailand.

**Keywords:** fall, fracture, meta-analysis, vitamin D, supplement

## Abstract

**Background:**

Vitamin D supplement is one of the current possible interventions to reduce fall and fracture. Despite having several studies on vitamin D supplement and fall and fracture reductions, the results are still inconclusive. We conducted a meta-analysis to examine the effect of vitamin D supplement in different forms and patient settings on fall and fracture.

**Methods:**

A systematic literature research was conducted in MEDLINE, EMBASE, and Cochrane Central Register of Controlled Trials to identify randomized controlled trials (RCTs) to compare the effects of vitamin D supplements on fall and fracture outcomes. Random-effect models were used to compute the weighted mean difference for continuous variables and the risk ratio for binary variables.

**Results:**

Forty-seven RCTs with 58,424 participants were identified reporting on fall outcome. Twenty-four of 47 studies with 40,102 subjects also reported fracture outcome. Major populations were elderly women with age less than 80 years. Overall, vitamin D supplement demonstrated a significant effect on fall reduction, RR = 0.948 (95% CI 0.914-0.984; *P* = .004, I^2^ = 41.52). By subgroup analyses, only vitamin D with calcium supplement significantly reduce fall incidence, RR = 0.881 (95% CI 0.821-0.945; *P* < .001, I^2^ = 49.19). Vitamin D3 supplement decreased incidence of fall but this occurred only when vitamin D3 was supplemented with calcium. Regarding fracture outcome, vitamin D supplement failed to show fracture lowering benefit, RR = 0.949 (95% CI 0.846-1.064; *P* = .37, I^2^ = 37.92). Vitamin D along with calcium supplement could significantly lower fracture rates, RR = 0.859 (95% CI 0.741-0.996; *P* = .045, I^2^ = 25.48).

**Conclusions:**

The use of vitamin D supplement, especially vitamin D3 could reduce incidence of fall. Only vitamin D with calcium supplement showed benefit in fracture reduction.

## 1 Introduction

In elderly, particularly female, falling is still the main burden and leading cause of mortality and morbidity. Therefore, falling is considered as a marker of poor health and declining function in both physical and social aspects.^[1]^ Fracture occurs in approximately 10% of falls depending on risk factors for falls, fall descent, fall impact, and bone strength.^[2,3]^ Therefore, fall prevention is necessary in terms of fracture prevention and reducing morbidity and mortality. Strategies to prevent fall include modification of environmental hazards, training paths, hip protectors, and appropriate use of support tools and balance exercises.^[4,5]^ However, the optimal type, duration, and intensity for these physical procedures necessary to prevent falls remains unclear and difficult to assess. Interventions to prevent fracture are also crucial. Nonetheless, long term effectiveness of various modalities to prevent fracture remains currently obscure.^[6–8]^

Supplement of vitamin D, one of the most commonly used pharmacologic agents, appears to be the easiest way to prevent and reduce fall and fracture. Although there have been several randomized controlled trials (RCTs) and meta-analyses regarding the role of vitamin D supplement on prevention of fall and fracture, the results are still inconclusive, reporting either having effectiveness or no benefit. The disparity in the results might be caused by differences in methodology, study quality, groups of population, calcium co-supplement, and details of vitamin D administration, including type, dose, frequency, and duration in these studies.^[8–15]^

Recently, a new recommendation from the US Preventive Services Task Force (USPSTF) 2018 opposed vitamin D supplement to prevent falls in community-dwelling adult 65 years or older with grade D recommendation as there is moderate or high certainty that vitamin D supplement has no net benefit or that the harms outweigh the benefits. Nevertheless, the 2018 USPSTF recommendation included only seven trials regarding vitamin D supplement with total population of only 7,531 subjects.^[14]^

As the results from previous studies were still not well-established, the present meta-analysis was conducted to re-assess the potential effectiveness of vitamin D supplement in different forms and methods of administrations on the incidences of falls and fractures.

## 2 Methods

### 2.1 Data sources and searches

We performed a MEDLINE literature search from 1990 to January 2019 to identify eligible studies using the Medical Subject Headings (MeSH) database search terms “Vitamin D”, “ergocalciferol”, “cholecalciferol”, “calcidiol”, “calcifediol”, “calcitriol”, “1-alpha hydroxylase” “25-hydroxyvitamin D”, “1,25-dihydroxyvitamin D” and “fall” or “fracture”. The search was limited to clinical trial and human studies. We also searched EMBASE and the Cochrane Central Register of Controlled Trials for completed studies using similar search terms. For this type of study, ethical approval is not required. This study protocol was registered in the PROSPERO: International Prospective Register of Systematic Reviews, registration number CRD42020158780.

### 2.2 Eligibility criteria

**Figure 1:**
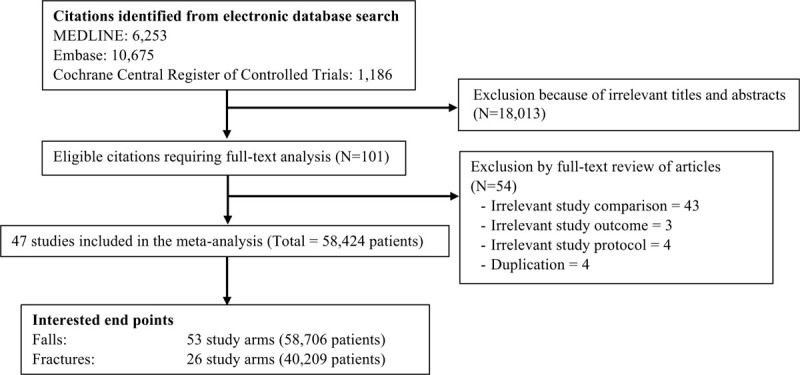
Flow diagram for selection of studies of Vitamin D supplement on falls.

We included RCTs comparing the effects of vitamin D supplement on fall and fracture related to fall. There were no restrictions on sample size, study duration, or language (Figure 1).

### 2.3 Study selection

Two authors (ST, AC) independently screened the titles and abstracts of all electronic citations, and full-text articles were retrieved for comprehensive review and independently re-screened. If there were any disagreements that did not have a conclusion, a third author (PS) would make a consensus.

### 2.4 Data extraction and quality assessment

The following data extracted from the RCTs were included in the study: country of origin, year of publication, study design, sample size, characteristics of population, duration of the intervention, percentage of women, number of faller, number of total fall, number of fracture patients with fracture, and number of total fracture, type of vitamin D supplementation, dosage and frequency of vitamin D administration. The authors of the included RCTs were contacted for the incomplete data via e-mail. Some missing data were also derived from other previous analyses if the authors were unreachable. Study quality was assessed with a modified version of the Jadad scale, which assesses randomization adequacy, blinding, and attrition, with higher scores reflecting better quality (score 0-5).^[16]^ The scores of 4-5 points would be categorized as a good study, 3 points as a fair study, and 0-2 as a poor study.

### 2.5 Data synthesis and statistical analysis

We compared data of fall and fracture using relative risk with an intention-to-treat analysis. We also used random-effect model meta-analyses to assess absolute change in continuous outcomes and risk ratio in dichotomous data. All pooled estimates are displayed with a 95% confidence interval (CI).^[17]^ A sensitivity analysis was performed to explore the impact of each individual study by limiting the included criteria and showing the precision of the results.

Existence of heterogeneity among effect sizes estimated by individual study was described with the I^2^ index and the chi-square test. An I^2^ index >50% was used to indicate medium-to-high heterogeneity.^[18]^ Publication bias was formally assessed using funnel plots and the Egger test, a test that determines asymmetry of the funnel plot, whereby a value of *P* < .05 indicates publication bias.^[18]^ The meta-analyses were performed with Comprehensive Meta-Analysis version 2.0 (www.meta-analysis.com; biostat, Englewood, NJ).

## 3 Results

### 3.1 Characteristics and quality of the studies

A total of 18,114 potentially relevant citations were identified and screened, and 101 articles were retrieved for detailed evaluation. Forty-seven of these studies^[19–65]^ fulfilled the eligibility criteria of inclusion in the meta-analysis and reported on fall incidence with total 58,424 patients. Twenty-four out of 47 studies^[19,21,22,25,27,29,30,32,36,38,40–43,45,46,48,49,51,54,57,61,62,64]^ with total 40,102 patients reported on fracture incidence (Fig. 1).

Among the 47 included studies reporting on incidence of fall, 42 studies reported raw data as an intention-to-treat analysis while 2 studies^[20,33]^ only reported on those with complete trials. The remaining 3 studies^[19,31,47]^ reported on relative risks without raw number for each intervention or treatment group. We extracted raw number of fall or relative risks from 44 out of 47 studied works. We could classify interventions into two main categories based on the type of vitamin D including 2 trials with vitamin D analogues, 7 trials with vitamin D2 supplement, 37 trials with vitamin D3 supplement, and 1 trial combined vitamin D2 and D3.

Among studies reporting on incidences of fracture, all of 24 studies reported raw data as an intention-to-treat analysis. All data in the studies were acquired directly from the original publication. There were 6 studies supplemented with vitamin D2, while 19 studies received vitamin D3 supplement. For both fall and fracture, there was one study with both forms of vitamin D supplement on the experimental groups. Therefore, we classified and analyzed data according to the form of vitamin D.

Characteristics of the individual trial were demonstrated in Table 1 and Table 2. The trials spanned for 28 years (1992-2019), varied in sample size (61 to 9,605 patients). The majority of the population was women aging less than 80 years. Twenty-nine studies out of forty-seven studies^[21,23,25,26,28,29,33,35,37,38,42,43,45–48,50–57,59,61–63,65]^ reporting on fall incidence had good quality (Jadad score 4-5), while the remaining 18 studies^[19,20,22,24,27,30–32,34,36,39–41,44,49,58,60,64]^ had fair quality (Jadad score 3). Fourteen studies out of 24 studies^[21,25,29,38,42,43,45,46,48,51,54,57,61,62]^ reporting on fracture incidence had good quality while the others^[19,22,27,30,32,36,40,41,49,64]^ had fair quality.

**Table 1:**
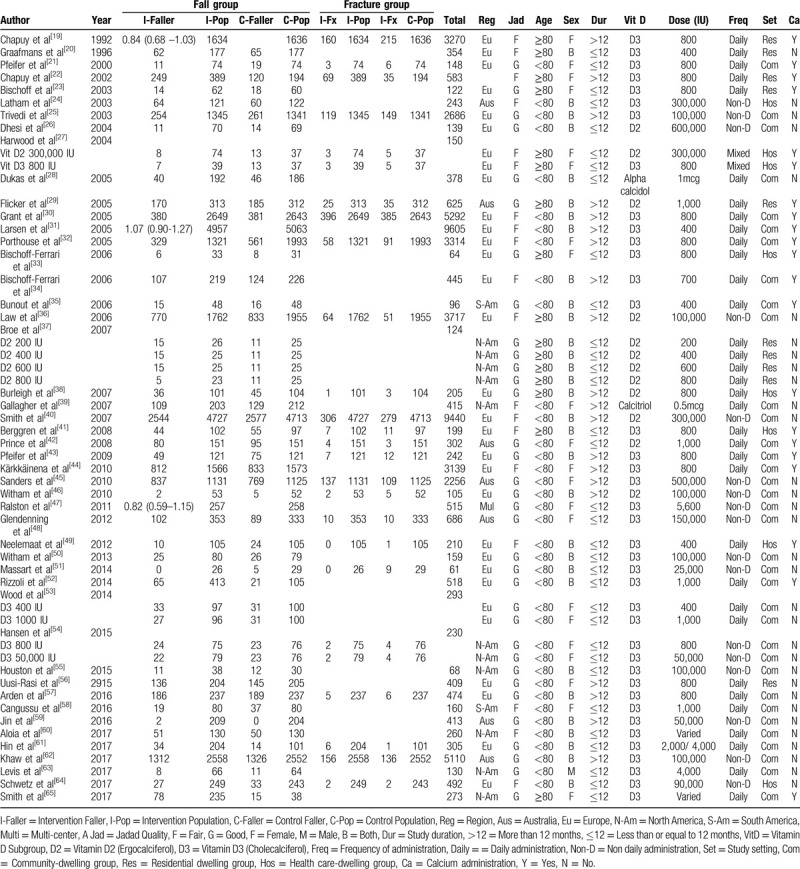
Characteristics of the studies focusing on fall and fracture outcomes included in the meta-analysis.

**Table 2:**
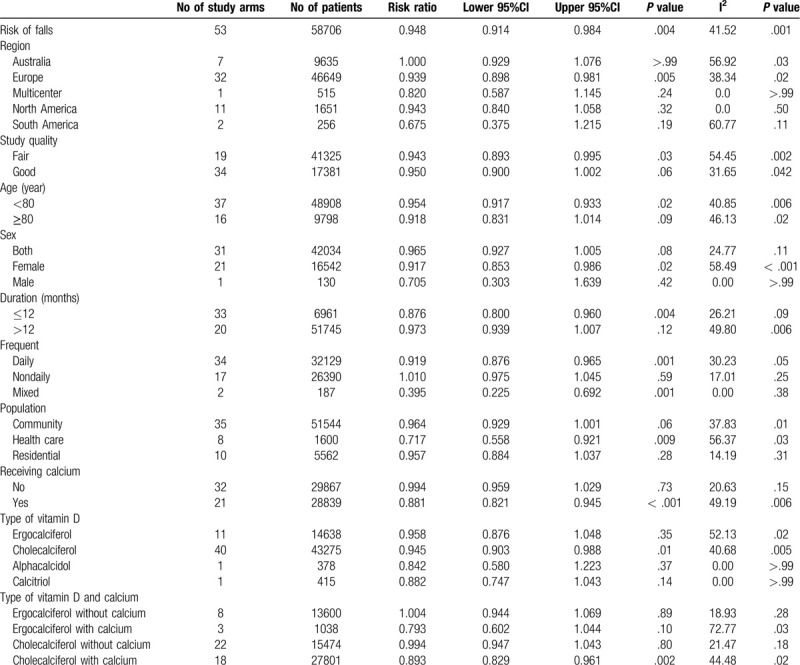
Subgroup analyses of meta-analysis for fall outcome.

### 3.2 Effect of vitamin D supplement on falling endpoints

Fifty-three study arms reporting on fall incidence underwent meta-analysis ^[19–65]^ Overall, vitamin D supplement statistically revealed benefit in reducing fall rate when compared with placebo, RR = 0.948 (95% CI 0.914-0.984; *P* = .004, I^2^ = 41.52).

### 3.3 Effect of vitamin D supplement on fracture endpoints

**Figure 2:**
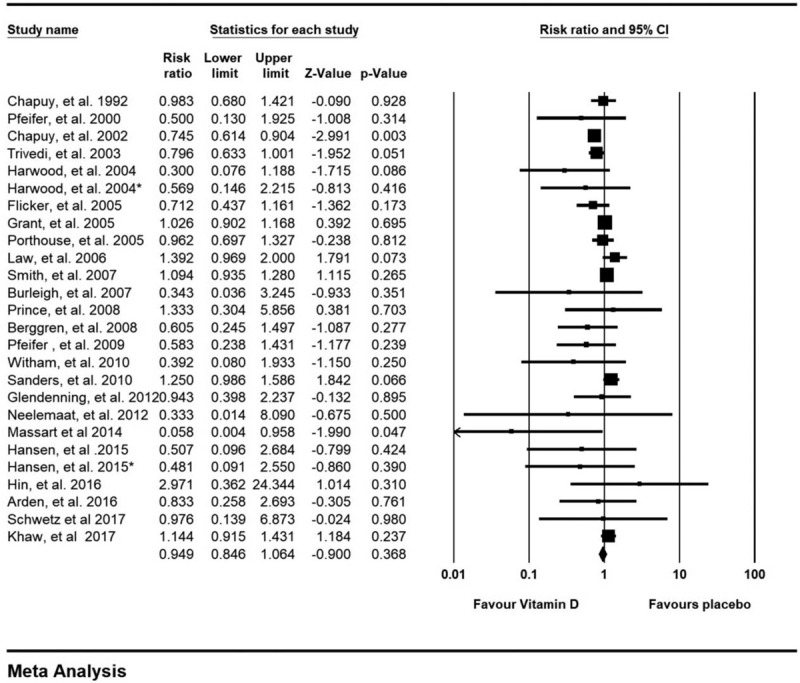
Forest plot on fracture outcomes.

Twenty-six study arms reported on the incidence of fracture.^[19,21,22,25,27,29,30,32,36,38,40–43,45,46,48,49,51,54,57,61,62,64]^ In our meta-analysis, vitamin D seemed to show benefit in reducing the incidence of fracture, but without statistical significance, RR = 0.949 (95% CI 0.846-1.064; *P* = .37, I^2^ = 37.92) (Fig. 2).

### 3.4 Investigations of heterogeneity

Table 2 and Table 3 reported the results of subgroup analyses exploring the risk ratio of fall and fracture outcomes stratified by nation of the study, study quality, mean age, sex, duration of supplement, type of vitamin D, frequency of supplement, group of population based on their habitats, and calcium supplement.

**Table 3:**
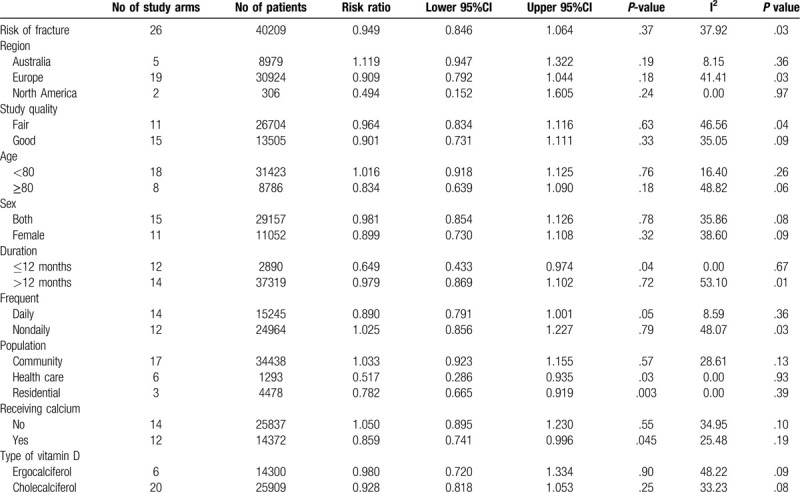
Subgroup analyses of meta-analysis for fracture outcome.

By subgroup analysis on fall outcomes (Table 2), studies from Europe appeared to have benefit on fall reduction, RR = 0.939 (95% CI 0.898 - 0.981; *P* = .005) whereas the studies from other regions including Australia, North America and South America did not provide the same benefit, RR = 1.000 (95% CI 0.929–1.076; *P* > .99), RR = 0.943 (95% CI 0.840 – 1.058; *P* = .32) and RR = 0.675 (95% CI 0.375 – 1.215; *P* = .19), respectively. Studies with fair study quality^[19,20,22,24,27,30–32,34,36,39–41,44,49,58,60,64]^ tended to support vitamin D supplementation, RR = 0.943 (95% CI 0.893–0.995; *P* = .03) while those with good study quality^[21,23,25,26,28,29,33,35,37,38,42,43,45–48,50–57,59,61–63,65]^ showed benefit without statistical significance, RR = 0.950 (95% CI 0.900 – 1.002; *P* = .06). Studies with mean ages less than 80 years old demonstrated that vitamin D could reduce incidence of falls but studies with mean ages over 80 years old could yield the same effects, but did not have statistical significance (RR = 0.954 (95% CI 0.917 – 0.933; *P* = .02) and RR = 0.918 (95% CI 0.831–1.014); *P* = .09, respectively))

By sex subgroup, vitamin D supplement could reduce falls in female participants, RR = 0.917 (95% CI 0.853 – 0.986; *P* = .02). Mixed male and female studies could not illustrate the same results, RR = 0.965 (95% CI 0.927–1.005; *P* = .08). Moreover, one study with only male participant did not show vitamin D benefit on fall prevention, RR = 0.705 (95% CI 0.303–1.639; *P* = .42). However, this result might not represent the male population because only one study was included in the analysis. Vitamin D supplement less than one year could reduce falls, whereas supplement more than one year could not present the same outcomes, RR = 0.876 (95% CI 0.800–0.960; *P* = .004) and RR = 0.973 (95% CI 0.939–1.007; *P* = .12). Daily vitamin D supplement demonstrated statistically significant effects on fall reduction, RR = 0.919 (95% CI 0.876–0.965; *P* = .001). However, non-daily vitamin D supplement seemed to increase fall incidence without statistical significance, RR = 1.010 (95% CI 0.975 – 1.045; *P* = .59). Studies including subjects from health care setting appeared to provide benefit from fall reduction, RR = 0.717 (95% CI 0.558–0.921; *P* = .009) while studies with participant's form community and residential cares seemed to show some benefit but without statistical significance, RR = 0.964 (95% 0.929–1.001; *P* = .06) and RR = 0.957 (95% 0.884–1.037; *P* = .28).

Comparing between subgroups with and without calcium supplement, subjects treated with calcium supplement has statistically significant fall reduction, RR = 0.881 (95% CI 0.821–0.945; *P* < .001) while those without calcium did not achieve the same outcomes, RR = 0.994 (95% CI 0.959–1.029; *P* = .73). Regarding, types of vitamin D supplement on incidence of falls, different results were observed. Only cholecalciferol seemed to show significant benefit, RR = 0.945 (95% CI 0.903–0.988; *P* = .01). Nonetheless, ergocalciferol tended to reduce falls without statistical significance, RR = 0.958 (95% CI 0.876–1.048; *P* = .35).

Effects of type of vitamin D combined with calcium supplement were also analyzed and revealed that only cholecalciferol with calcium supplement could significantly reduce falls, RR = 0.893 (95% CI 0.829–0.961; *P* = .002). However, ergocalciferol with or without calcium and cholecalciferol without calcium did not attain statistically significant outcomes, RR = 0.793 (95% CI 0.602–1.044; *P* = .10), RR = 1.004 (95% CI 0.944–1.069; *P* = .89) and RR = 0.994 (95% CI 0.947–1.043; *P* = .80). In sensitivity analysis based on high dose of daily vitamin D supplement defined by equal and more than 800 international units (IU) per day, Treatment with high dose vitamin D could decrease incidences of falls, RR = 0.884 (95% CI 0.830 - 0.943; *P* < .001).

In term of fracture risk (Table 3), by subgroup analysis, participants from different regions consisting of Australia, Europe and North America could not significantly exhibit the benefit of vitamin D supplement on fracture reduction, RR = 1.119 (95% CI 0.947–1.322; *P* = .19), RR = 0.909 (95% CI 0.792 – 1.044; *P* = .18) and RR = 0.494 (95% CI 0.152 – 1.605; *P* = .24), respectively. Both good study quality^[21,25,29,38,42,43,45,46,48,51,54,57,61,62]^ and fair study quality^[19,22,27,30,32,36,40,41,49,64]^ seemed to reduce fracture incidences without statistical significance, RR = 0.901 (95% CI 0.731 – 1.111; *P* = .33) and RR = 0.964 (95% CI 0.834–1.116; *P* = .63). Ages less or over 80 years did not demonstrate significant results on fracture reduction, RR = 1.016 (95% CI 0.918–1.125; *P* = .76) and RR = 0.834 (95% CI 0.639 – 1.090; *P* = .18). Studies from female sex and both sexes showed fracture reduction without statistical significance, RR = 0.899 (95% CI 0.730–1.108; *P* = .32) and RR = 0.981 (95% CI 0.854–1.126; *P* = .78). Less than one year of vitamin D supplement could reduce fractures whereas those receiving supplement more than one year could not yield the same outcomes, RR = 0.649 (95% CI 0.433–0.974; *P* = .04) and RR = 0.979 (95% CI 0.869–1.102; *P* = .72).

Daily vitamin D supplement tended to reduce fracture incidences without statistical significance, RR = 0.890 (95% CI 0.791–1.001; *P* = .05). On the contrary, non-daily supplement seemed to increase risk of fractures, RR = 1.025 (95% CI 0.856–1.227; *P* = .79). Studies from health care and residential cares showed statistical significance, RR = 0.517 (95% CI 0.286–0.935; *P* = .03) and RR = 0.782 (95% CI 0.665–0.919; *P* = .003). However, studies from community settings failed to show benefit on fracture reduction, RR = 1.033 (95% CI 0.923–1.155; *P* = .57). Subgroup with calcium supplement showed benefit on fracture reduction, RR = 0.859 (95% CI 0.741–0.996; *P* = .045) whereas studies without calcium supplement tended to increase fracture incidences, RR = 1.050 (95% CI 0.895–1.230; *P* = 0.55). Both ergocalciferol and cholecalciferol seemed to reduce fracture incidence without statistical significance, RR = 0.980 (95% CI 0.720–1.334; *P* = .90) and RR = 0.928 (95% CI 0.818–1.053; P = 0.25) respectively. In sensitivity analysis based on high dose defined by vitamin D supplement equal and more than 800 IU per day, high dose vitamin D could reduce incidences of fracture, RR = 0.883 (95% CI 0.780–0.999; *P* = .048).

### 3.5 Assessment of publication bias

The funnel plot for the outcomes of fall and fracture in the studies included in the meta-analysis was asymmetric and the Egger test was significant (*P* < .001, and *P* = .03), respectively, suggesting susceptibility to publication bias.

## 4 Discussion

The present meta-analysis included more than 58,000 participants who were mainly elderly female from all over the world, mostly Europe, and were principally community-dwelling group. With respect to fall aspect, the overall results demonstrated that vitamin D supplement provided significant benefit on preventing incidence of fall. By subgroup analysis, only vitamin D3 could exhibit significant effects on fall reduction and this developed when was co-supplemented with calcium. Daily vitamin D supplement, duration of supplement less than 12 months, European population, and health care-dwelling population were related with reduction in fall incidence. The fracture lowering benefit of vitamin D supplement occurred only when was co-administered with calcium.

Regarding fall outcome, the comparative details of all previous meta-analyses, the present study, and the 2018 USPSTF recommendation were illustrated in Table 4 .^[9–15]^ Among earlier meta-analyses, the studies by Bolland et al and Murad et al included much larger number of RCTs and participants than the remaining works.^[12,15]^ The meta-analysis by Bolland et al in 2018, which included 37 RCTs with 34,144 participants, focused on vitamin D monotherapy, discarded studies using vitamin D analogues, and excluded studies which compared between vitamin D plus calcium and placebo.^[15]^ The meta-analysis by Murad et al in 2011, which contained 26 RCTs with 45,782 subjects, were quite similar to the present study but lesser number of included RCTs as well as participants.^[12]^ Furthermore, issues regarding method of vitamin D administration and duration of treatment were not assessed in such study. Of note, the 2018 USPSTF recommendation, which included only 7 RCTs with 7,532 participants, concentrated on only vitamin D3 or active form of vitamin D3 and on community dwelling setting.^[14]^ Therefore, the present meta-analysis included the largest number of participants. Moreover, the current meta-analysis examined more extensive aspects than all previous works (Table 4 ).

**Table 4:**
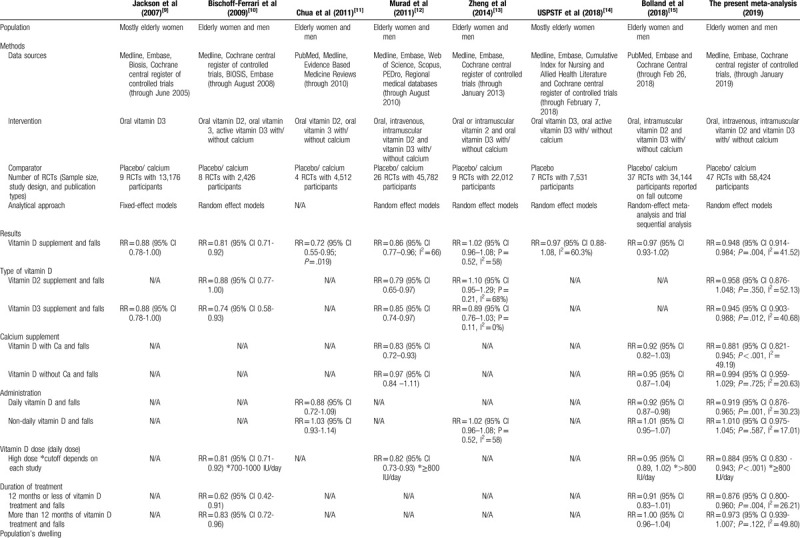
Comparison between previous meta-analyses and the present study on fall outcome.

**Table 4 (Continued):**
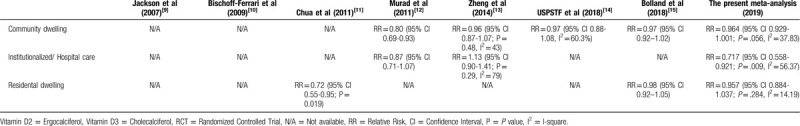
Comparison between previous meta-analyses and the present study on fall outcome.

The results from the study by Murad et al were in agreement with the present meta-analysis that only vitamin D with calcium supplement provided fall lowering benefit.^[12]^ On the contrary, the meta-analysis by Bolland et al and the 2018 USPSTF recommendation yielded non-significant fall lowering outcomes of vitamin D with or without calcium.^[14,15]^ Such discrepancy might be caused by study-related issues in each study, as stated above, resulting in smaller number of participants and imprecise results. The present meta-analysis illustrated the fall lowering benefit of vitamin D supplement in only health care dwelling but not in community-dwelling and residential-dwelling. In this regard, the 2018 USPTSF recommendation also stated that there were no fall decreasing benefit for vitamin D supplement in the community-dwelling group while the study by Murad et al observed favorable results in such group.^[14]^

From our analysis, vitamin D3 could significantly reduce fall. The result in the previous meta-analysis by Bischoff-Ferrari et al^[10]^ concurred with the present study. However, the study by Murad et al reported that both vitamin D2 and D3 could decrease fall incidence.^[12]^ On the other hand, the study by Zheng et al failed to show beneficial effect on both groups.^[13]^ These varied results across studies might be due to different numbers of studies included in each analysis. One feasible reason why vitamin D3 seemed to have greater benefit on fall prevention than vitamin D2 might be due to its superior ability to increase serum vitamin D level which was observed in some studies^[66–68]^ and those with higher vitamin D level tended to experience less falls.^[69]^

Daily supplement of vitamin D seemed to prevent falls while intermittent supplement increased fall risks without statistical significance. A recent study by Bolland et at^[15]^ also reported similar outcome. Other analyses by Chua et al and Zheng et al also demonstrated that non-daily vitamin D supplement raised fall incidences.^[11,13]^ There is no clear reason why non-daily vitamin D supplement increased risk of falls. However, intermittent supplements were usually given in very high doses of vitamin D which were believed to be the causes of increasing falls. Some studies proposed a U- or J-curve phenomena of vitamin D dose but some suggested that the effect of intermittent high dose of vitamin D might be mediated towards vitamin D receptor in the central nervous system, leading to increased falls.^[11,13,70]^

That only vitamin D3 with calcium supplement could reduce fall incidence would underscore the contributory role of both agents in fall lowering benefit. (Table 2) The mechanisms of vitamin D with calcium supplement in attenuating fall incidence are still unestablished. However, vitamin D and calcium supplement could possibly affect calcium homeostasis, increase muscle strength, improve body sway and decrease parathyroid hormone secretion and bone resorption, leading to reduced risk of falling.^[21,23,71–74]^ Inadequate vitamin D and calcium intake could raise serum parathyroid hormone (PTH), leading to bone turn over and bone loss. Treatment with vitamin D and calcium supplement could decrease serum PTH and improve body sway. Less body sway might lead to lower fall incidence.^[21,75,76]^ In 2014, a meta-analysis, which included 30 RCTs with 5,615 participants, showed a small but significant positive effect of vitamin D supplement on global muscle strength with a standardized mean difference (SMD) of 0.17 (95% CI 0.03–0.31; *P* = .02). However, no significant effect was found on muscle mass or muscle power. In subgroup analysis, muscle strength was significantly improved in people who presented with serum 25-hydroxyvitamin D (25-OHD) lower than 12 ng/dL and in those who were older than 65 years.^[77]^ In a trial focusing on neuromuscular and psychomotor function in the elderly, people who experienced falling had weaker quadriceps, slower functional performance, slower reaction times, and impaired postural stability compared with healthy age-matched subjects. The subjects who fell also had serum 25-OHD less than 12 ng/dL.^[78]^

High dose of daily vitamin D supplement (more than 800 IU/day) appeared to decrease falling incidences. This finding was supported by a study by Murad et al which demonstrated that higher dose of vitamin D could reduce falls.^[12]^ In contrast, the meta-analysis by Bolland et al did not show the same results.^[15]^ This could be explained by different cutoff values of high or low vitamin D dose defined by each study which could affect the numbers of participants analyzed in each group.

Studies with less than 12-month intervention had positive effects on fall prevention. Previous meta-analyses by Bischoff-Ferrari et al and Bolland et al^[10,15]^ were in agreement with the present works. Nonetheless, the present study and Bolland et al revealed that longer interventions had insignificant results.^[15]^ Theoretically, longer supplements should also reduce fall incidences. Indeed, we only analyzed data by numbers of fallers and longer follow-up periods could result in increasing numbers of the fallers. To correct this effect, the numbers of total falls should be further carefully studied.

By study locations, this meta-analysis exhibited the benefit of vitamin D supplement in health care setting significantly and also demonstrated the benefit on community and residential subjects without statistical significance. The results from previous studies were varied possibly due to different categorizations among each study.^[11–15]^

With respect to fracture issue, the overall results in the present meta-analysis illustrated that vitamin D failed to yield fracture reducing benefit. Subgroup analysis revealed that vitamin D2 and D3 could not reduce fracture rate. Only vitamin D with calcium supplement significantly lowered the incidence of fracture. Furthermore, higher dose of vitamin D supplement (more than 800 IU/day) could also significantly reduce incidences of fractures. Nevertheless, both daily and non-daily vitamin D supplement could not attenuate fracture rate. Only health care-dwelling and residential care-dwelling groups gained fracture lowering benefit from vitamin D supplement. The underlying mechanisms of vitamin D with calcium supplement in fracture attenuating effect are still inconclusive. Vitamin D helps control calcium absorption from the small intestines and work together with PTH to maintain calcium homeostasis between the blood and bones. Insufficient vitamin D acquirement can lead to impaired calcium absorption from diet. As a result, calcium from skeletal storage is used and this could weaken the bones.^[8]^ Vitamin D and calcium insufficiencies were also found to induce hyperparathyroidism, leading to increase bone turn over, bone loss, and fracture.^[19,22,75]^ Treatment with vitamin D and calcium supplement showed decreased serum PTH, increased bone mineral density (BMD), and reduce fracture rates.^[19,22,79,80]^ The improvement of bone density concurrently with muscle strengthening, and lower rates of falls, might finally result in lessening fracture incidence. Despite these plausible mechanisms, in a large RCT involving 36,282 postmenopausal women, hip bone density was found higher in the vitamin D supplement group but there was no significant reduction in hip fracture.^[81]^ However, our results found that subgroup analysis with female participants tended to reduce fracture incidence without statistical significance. This might be explained by the fact that fractures could result from multifactorial factors and the higher bone mass density from vitamin D supplement might not be sufficient to reach the essential level to prevent fractures.

The strengths of our meta-analysis include 1) the literature has been extensively performed by blinded pairs of reviewers, resulting in large number of trials and populations on fall and fracture and 2) all aspects regarding vitamin D supplement and prevention of fall and fracture have been carefully analyzed. Admittedly, there are some limitations for our meta-analysis to be considered. The heterogeneity of the studies and publication bias are the concerns of the issues. The study designs were different according to their primary objectives and outcomes. Population from each study was not the same. Some studies focused on post-menopausal women whereas some included both sexes. The setting of population ranged from community-dwelling elderly to hospitalized patients. Vitamin D supplement was given in different forms, dosage, and duration. However, we tried to categorize the characteristics from each study and reported as subgroup analyzes. Since, there was no report on dietary intake, each patient might receive different amount of daily vitamin D and this could affect the results. The studies did not categorize fall as injurious or non-injurious fall which were important outcomes to predict recurrent fall and physical function. Moreover, fractures in the studies were reported separately and could not be implied to be the results of falls.

In conclusion, this current meta-analysis of 47 RCTs encompassing 58,424 patients demonstrated that the use of vitamin D supplement, especially vitamin D3 could reduce incidence of fall. Only vitamin D with calcium supplement showed benefits in fracture reduction. We recommend daily vitamin D3 supplement with equal and more than 800 IU per day combined with calcium to prevent falls. In addition, daily vitamin D supplement over 800 IU per day co-administered only with calcium was also advised for fracture prevention.

## Author contributions

**Conceptualization:** Saran Thanapluetiwong, Somchai Eiam-Ong, Paweena Susantitaphong.

**Data curation:** Saran Thanapluetiwong, Api Chewcharat, Paweena Susantitaphong.

**Formal analysis:** Kullaya Takkavatakarn, Kearkiat Praditpornsilpa, Somchai Eiam-Ong, Paweena Susantitaphong.

**Investigation:** Saran Thanapluetiwong, Api Chewcharat, Kullaya Takkavatakarn, Kearkiat Praditpornsilpa, Somchai Eiam-Ong, Paweena Susantitaphong.

**Methodology:** Saran Thanapluetiwong, Api Chewcharat, Kullaya Takkavatakarn, Kearkiat Praditpornsilpa, Somchai Eiam-Ong, Paweena Susantitaphong.

**Project administration:** Saran Thanapluetiwong, Api Chewcharat, Paweena Susantitaphong.

**Resources:** Saran Thanapluetiwong, Api Chewcharat.

**Supervision:** Kearkiat Praditpornsilpa, Somchai Eiam-Ong, Paweena Susantitaphong.

**Validation:** Kullaya Takkavatakarn, Kearkiat Praditpornsilpa, Somchai Eiam-Ong, Paweena Susantitaphong.

**Writing – original draft:** Saran Thanapluetiwong, Api Chewcharat.

**Writing – review & editing:** Saran Thanapluetiwong, Api Chewcharat, Kullaya Takkavatakarn, Kearkiat Praditpornsilpa, Somchai Eiam-Ong, Paweena Susantitaphong.
